# Livin is protective in UVB‐induced skin photodamage by regulating keratinocyte activation and inflammatory responses

**DOI:** 10.1111/jcmm.18124

**Published:** 2024-02-08

**Authors:** Kaijie Wang, Xiaolan You, Zhenri Qu, Delu Che, Xianwei Cao

**Affiliations:** ^1^ Department of Dermatology, The 1st affiliated hospital, Jiangxi Medical College Nanchang University Nanchang China; ^2^ Department of Dermatology The Second Affiliated Hospital of Xi'an Jiaotong University Xi'an China

**Keywords:** inflammatory response, keratinocyte, Livin, skin photodamage, UVB

## Abstract

UVB radiation can lead to skin photodamage, which might arise from keratinocyte (KC) activation. Nuclear factor kappa B (NF‐κB) assumes an essential function in the context of UVB‐triggered skin photodamage. Initiating the NF‐κB cascade leads to the release of inflammatory factors from KCs. Livin can modulate both KC activation and function, yet it remains uncertain whether and how Livin regulates KC activation induced by UVB. To explore the involvement of Livin in UVB‐triggered skin photodamage and its impact on skin damage through NF‐κB activation. Immunofluorescence staining was used to analyse the expression of Livin in individuals with skin photodamage and in mice treated with UVB radiation. KC‐specific Livin knockout (Livin^ΔKC^) mice and HaCaT cells with Livin knockdown were employed to examine the function of Livin in regulating KC activation induced by UVB radiation. Additionally, the impact of Livin on the NF‐κB cascade during KC activation was confirmed via western blot analysis. In patients with skin photodamage, UVB‐treated mice and HaCaT cells, Livin expression was reduced in KCs. Livin^ΔKC^ mice displayed heightened sensitivity to UVB radiation, resulting in more pronounced skin damage and inflammatory responses compared to the control Livin^fl/fl^ mice. Following UVB exposure, both Livin^ΔKC^ mice and Livin‐knockdown HaCaT cells released elevated levels of cytokines compared to their respective controls. Moreover, the UVB‐induced activation of NF‐κB in HaCaT cells was significantly enhanced following Livin knockdown. Our findings propose that Livin within KCs could contribute to reducing UVB‐induced skin photodamage by regulating the NF‐κB pathway.

## 1 INTRODUCTION

The skin acts as the primary defence against a variety of external stimuli. Numerous factors, including physical and mechanical insults, chemical agents, and pathogenic microorganisms, can induce skin damage, among which ultraviolet (UV) damage stands out as a prevalent factor in everyday experiences.^1^, ^2^ Excessive UV light can result in various skin injuries, including sunburn, erythema, photoaging and photocarcinogenesis.^3^, ^4^ Ultraviolet light‐induced pathological processes encompass apoptosis, the activation of numerous inflammatory signal transduction mechanisms and irregularities in genes regulating cytokine activity.^5^

The primary spectra that induce skin photodamage encompass Type A (315–340 nm) and Type B (280–320 nm) ultraviolet radiation. UVA radiation is capable of strong penetration and can reach the subcutaneous tissue, affecting the structures of both the dermis and epidermis.^6^ Although possessing a shorter wavelength, UVB radiation is primarily taken in by KCs situated in the epidermis; however, it can lead to rapid skin redness and swelling, ultimately culminating in skin photodamage. The degree of damage caused by UVB radiation is hundreds of times more potent than that induced by an equivalent dose of UVA radiation.^7^ Prolonged exposure to UVB radiation can lead to thickening and roughening of the epidermis and dermis. In severe cases, the skin becomes lax, folds deepen and coarsen, localized hyperpigmentation arises, and various benign or malignant tumours may develop.^8^ Hence, type B ultraviolet radiation is a significant contributor to skin photodamage.

The Livin gene, which is associated with the inhibitor of apoptosis protein (IAP) family, is 4.6 kb in size and encodes a protein with a weight of 31 kDa. It is situated on chromosome 20q13.3 in humans. Its expression has the potential to directly impede the proliferation and spread of cancer cells.^9^, ^10^ A recent investigation has shown that Livin plays a role in KC activation during the inflammatory response to psoriasis. Decreased Livin expression has been linked to the increased release of cytokines in KCs.^11^ Nevertheless, there have been limited investigations into the role of Livin in keratinocytes and its impact on the development of UVB‐induced skin photodamage.

Keratinocytes are the primary targets of UVB radiation within the epidermis. Nearly all UVB radiation is absorbed by epidermal KCs,^12^ and an excess of UVB radiation can induce gene mutations, DNA damage, oxidative stress, inflammation and other effects.^13^, ^14^ Exposure to UVB radiation can initiate the stimulation of KCs by triggering NF‐κB (Nuclear factor kappa B), which subsequently prompts the release of cytokines including interleukin (IL)‐1β, IL‐6 and tumour necrosis factor‐α (TNF‐α). This process triggers skin inflammation and consequently results in skin photodamage.^15^ However, whether NF‐κB is engaged in KC activation regulated by Livin remains uncertain.

This study aimed to explore how Livin functions in the regulation of KC activation and the release of cytokines during UVB‐induced skin photodamage. Our findings could offer valuable targets for addressing UVB‐induced skin photodamage.

## 2 MATERIALS AND METHODS

### 2.1 Immunoassays

Anti‐Livin antibody and anti‐Ly6G antibody were acquired (Thermo Fisher Scientific, Inc.). ELISA kits for mouse and human Livin, IL‐1β, IL‐6, TNF‐α, S100A3, S100A7, BD1, keratin 14 and keratin 17 were acquired (Enzyme‐linked Biotechnology Co., Ltd). Both companies are situated in Shanghai, China.

### 2.2 Examination of Livin protein levels in UVB‐treated HaCaT cells and human skin specimens

Samples of photodamaged skin (*n* = 8) and healthy skin derived from normal skin adjacent to keloid lesions (*n* = 8) were gathered from the Department of Dermatology at The Second Affiliated Hospital of Xi'an Jiaotong University. The collection of human skin specimens adhered to our institutional protocols and was endorsed by the Ethics Committee of Xi'an Jiaotong University (ref. no. 2022–1040). Within this research study, all enrolled patients provided informed consent. Subsequently, cross sections (4 μm) of the photodamaged skin were labelled by immunofluorescence staining utilizing an anti‐Livin antibody. Under UVB exposure, Livin mRNA expression levels in HaCaT cell samples were analysed using RNA‐seq data sets (GSE198792 and GSE201850), which downloaded from the GEO database. Additionally, HaCaT cells encountered different levels of UVB radiation (25, 50, 100 mJ/cm^2^), and Livin protein concentrations were evaluated using an ELISA kit and western blotting.

### 2.3 Mice

Livin^flox/flox^ mice (Livin^fl/fl^; C57BL/6 background; Stock No. S‐CKO‐10628) and K5‐CreERT2 transgenic mice (C57BL/6 background; Stock No. C001054) were procured from Cyagen Biosciences (Suzhou, China) and crossbred to yield K5‐Cre^ERT2+^/Livin^fl/fl^ mice. The generation of mice with a KC‐specific Livin knockout (K5‐Cre^ERT2+^/Livin^−/−^, referred to as Livin^ΔKC^) was achieved through tamoxifen induction. Livin expression in mice was evaluated using ELISA and RT‐qPCR (real‐time quantitative). Figure S1 presents the corresponding data. Approval for the experimental procedures concerning the mice was granted by the Animal Ethics Committee at Xi'an Jiaotong University in Xi'an, China (Approval Number: XJTU 2023–1538).

### 2.4 UVB‐induced skin photodamage mice

The backs of Livin^fl/fl^ and Livin^ΔKC^ mice were shaved, and the skin on their backs was divided into two sections using light‐tight tape. Following exposure to 250 and 500 mJ/cm^2^ UVB radiation, the mice were euthanized 24 h later by CO_2_ inhalation. Skin tissue samples (*n* = 8/group) were obtained.

### 2.5 RNA‐seq

Total RNA from UVB‐treated skin samples of Livin^fl/fl^ and Livin^ΔKC^ mice was extracted using TRIzol following the manufacturer's instructions. RNA‐seq analysis was conducted by Berry Genomics Corporation in Beijing, China. The viability of this configuration for experimentation was calculated using DESeq2 based on NCBI BioProject ID PRJNA993569. Utilizing R, the analysis of sequencing data and Gene Ontology (GO) and Kyoto Encyclopedia of Genes and Genomes (KEGG) assessments were undertaken (https://www.r‐project.org/).

### 2.6 Histology

Following fixation in 4% paraformaldehyde, mouse skin samples were embedded in paraffin. Neutrophils were labelled using immunohistochemical staining with the anti‐Ly6G antibody. Thin cross‐sections (4 μm) of the photodamaged skin were stained using haematoxylin and eosin (H&E).

### 2.7 Cytokines and keratin assay

Skin samples from mice were collected in a chilled conditions using RIPA buffer supplemented with a mixture of phosphatase inhibitors and 10% protease inhibitors from Roche Diagnostics. Subsequently, the protein content was assessed utilizing NanoDrop Microvolume Spectrophotometers (Thermo Fisher Scientific Inc., Shanghai, China). ELISA kits for mouse IL‐1β, IL‐6, TNF‐α, S100A3, S100A7, BD1, keratin 14 and keratin 17 were utilized to assess cytokine release and changes in keratin levels. The protein levels of cytokines and keratin were normalized by dividing by the total protein concentration.

### 2.8 Cell lines

Livin‐knockdown HaCaT cells and Livin‐overexpressing HaCaT cells were derived from our prior experiments; HaCaT refers to a keratinocyte cell line.^11^ Negative control (NC‐HaCaT), knockdown HaCaT and overexpression HaCaT cells were produced through the utilization of lentiviral vectors based on HIV‐1. The three cell types were cultured in RPMI 1640 medium with a 1:100 concentration of penicillin–streptomycin and enriched with 10% FBS. Livin expression was evaluated through ELISA and RT‐qPCR analyses (Figure S2).

### 2.9 Cytokine release and keratin expression analysis

In a 12‐well plate, a total of 1 × 10^5^ NC HaCaT cells, knockdown HaCaT cells and overexpression HaCaT cells were seeded and incubated at 37°C with 5% CO_2_ for 24 h. Afterwards, the medium was removed, and 300 μL of PBS was placed in the 12‐well plate, followed by exposure of the three cell types to 25, 50 and 100 mJ/cm^2^ UVB radiation. After removing the PBS, 1 mL of RPMI 1640 medium was added to each well. Cells were then cultured for an additional 24 h at 37°C with 5% CO_2_. Subsequently, cell cultures were harvested, and total proteins were isolated using the RIPA method. Human IL‐1β, IL‐6, TNF‐α, S100A3, S100A7, BD1, keratin 14 and keratin 17 were quantified using ELISA kits.

### 2.10 Signalling pathway analysis

The protein concentrations were detected using NanoDrop microvolume spectrophotometers from Thermo Fisher Scientific, Inc. (Shanghai, China). In a six‐well plate, 1 × 10^5^ NCHaCaT and Livin knockdown HaCaT cells were inoculated and cultured at 37°C with 5% CO_2_ for 24 h. Subsequently, the cells were subjected to 100 mJ/cm^2^ UVB radiation. Untreated cells were employed as the negative control. Total proteins from various groups of cells were isolated using RIPA buffer supplemented with a mixture of phosphatase inhibitors and 10% protease inhibitors. After denaturation through boiling with the sample buffer, the protein lysates underwent 10% SDS‐PAGE and were then transferred to PVDF membranes. The membrane‐blocking step involved incubating the membranes in a blocking solution (5% skim milk in TBS‐T) for 2 h at room temperature. The primary antibodies, which included anti‐P‐P65, anti‐P65, anti‐IKK, anti‐P‐IKK, anti‐P‐IκB, anti‐IκB and anti‐GAPDH and were obtained from OriGene Technologies, Inc. (Rockville, MD, USA), were incubated on the membranes at 4°C overnight. The dilution ratios of the primary antibodies strictly followed the provided instructions. After washing thrice with TBS‐T, the were incubated with secondary antibodies at a dilution of 1: 20,000 in TBS‐T for a duration of 1 h at 37°C. To visualize the protein bands, an enhanced chemiluminescence kit was employed. The outcomes were then assessed using the Image‐Pro Plus 5.1 software in conjunction with a Lane 1DTM transilluminator.

To demonstrate the effect of Livin on NF‐κB activation, an NF‐κB inhibitor was used in vivo (30 μM) and in vitro (100 mg/kg). After treatment with 100 mJ/cm^2^, both NC HaCaT cells and knockdown HaCaT cells exhibited decreased levels of IL‐1β, IL‐6, TNF‐α, S100A3, S100A7 and BD1 release, as well as reduced expression levels of keratin 14 and keratin 17, compared to the conditions without the NF‐κB inhibitor.

### 2.11 Statistical analysis

The data are displayed as the mean ± SEM. For two‐group comparisons, data were assessed through a nonpaired Student's *t*‐test. For data from more than two groups, one‐ or two‐way ANOVA followed by multiple group comparison was used. Statistical significance in the data comparisons was evaluated through an independent‐samples analysis of variance using the SPSS software. A *p* value below 0.05 was considered indicative of statistical significance.

## 3 RESULTS

### 3.1 Excessive UVB radiation significantly downregulated Livin expression

**FIGURE 1:**
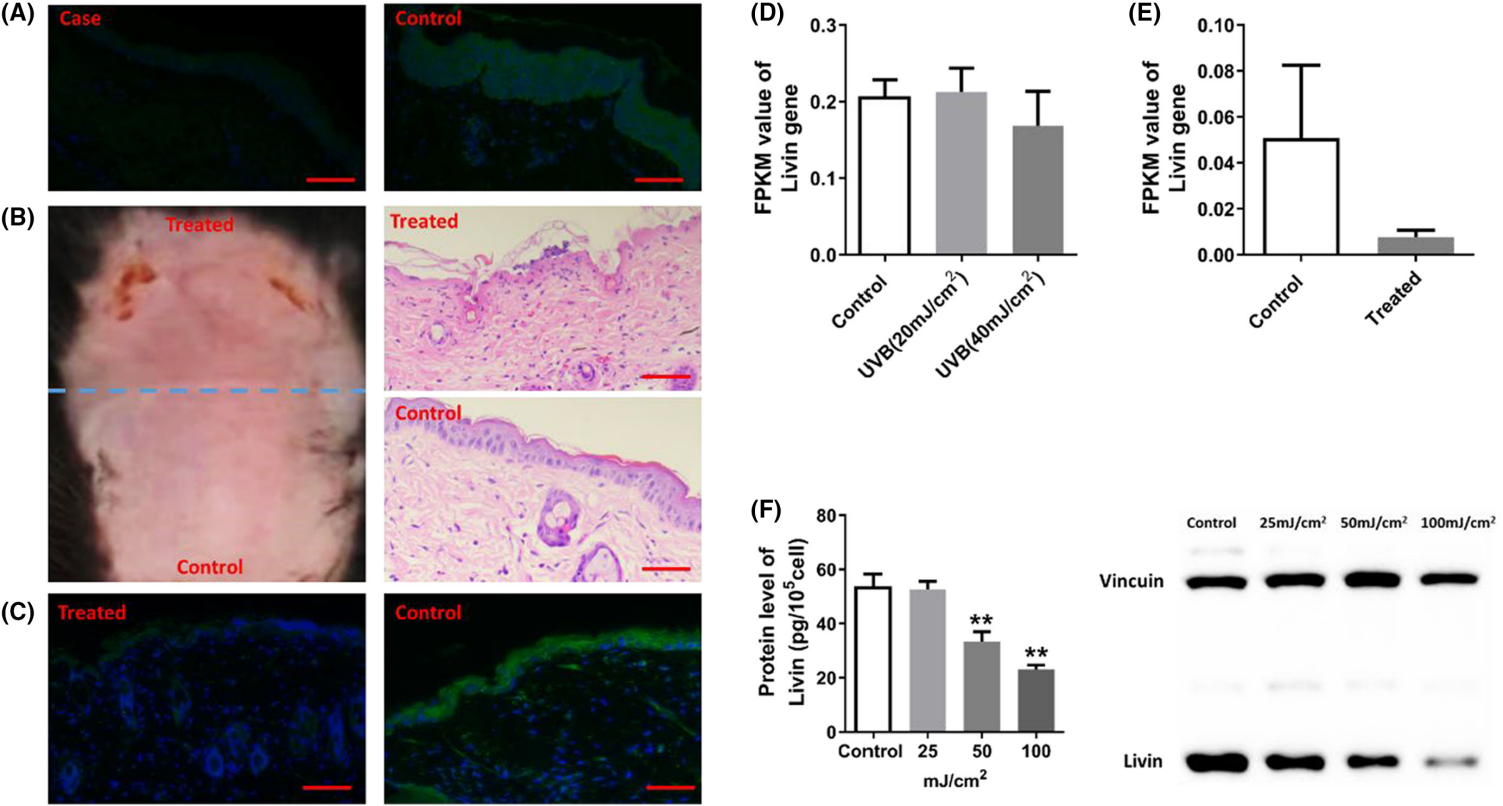
Livin expression was downregulated in KCs following UVB treatment. (A) Livin expression was significantly decreased in skin photodamage (Case) patient skin samples compared to healthy samples (Control), as demonstrated through immunofluorescence staining (*n* = 8, 200×). (B) UVB exposure of 500 mJ/cm^2^ induced skin photodamage in Livin^fl/fl^ mice, as evidenced by pathological analysis using haematoxylin and eosin staining (200×). The division between the treated and control areas is indicated by the blue dashed line. (C) Following treatment with 500 mJ/cm^2^ UVB, Livin expression in the epidermis of the treated group was downregulated in comparison to the control group, as indicated by immunofluorescence staining (*n* = 8, 200×). (D, E) The amount of Livin mRNA was analysed by RNA‐seq in UVB‐treated HaCaT cells. mRNA fragment quantities were standardized using the fragments per kilobase of transcript per million mapped reads (FPKM) (*n* = 3/group, biological replicates). (F) Livin protein levels significantly decreased after UVB treatment and were assessed using ELISA and western blotting (*n* = 3/group, biological replicates). **p < 0.01.

Livin expression was assessed through immunofluorescence staining in skin lesions from patients with skin photodamage (case group) and healthy individuals (control group). The findings pointed to a notably reduced Livin concentration in the case group in comparison with the control group (Figure 1A). In mice, following exposure to 500 mJ/cm^2^ UVB radiation, which induced skin photodamage in Livin^fl/fl^ mice (Figure 1B), Livin expression was decreased in the epidermal tissues of the group exposed to UVB radiation, in contrast to the control group (Figure 1C). Moreover, analysis of RNA‐sequencing (RNA‐seq) data from UVB‐treated HaCaT cells revealed that UVB radiation reduced Livin mRNA levels in these cells (Figure 1D,E). The level of Livin in HaCaT cells exhibited a dose–response relationship with UVB exposure. To determine Livin protein expression, HaCaT cells were subjected to three different doses of UVB radiation. As the UVB dose increased, there was a notable reduction in Livin protein levels (Figure 1F).

### 3.2 Livin^ΔKC^ mice were more sensitive to skin damage caused by UVB exposure

**FIGURE 2:**
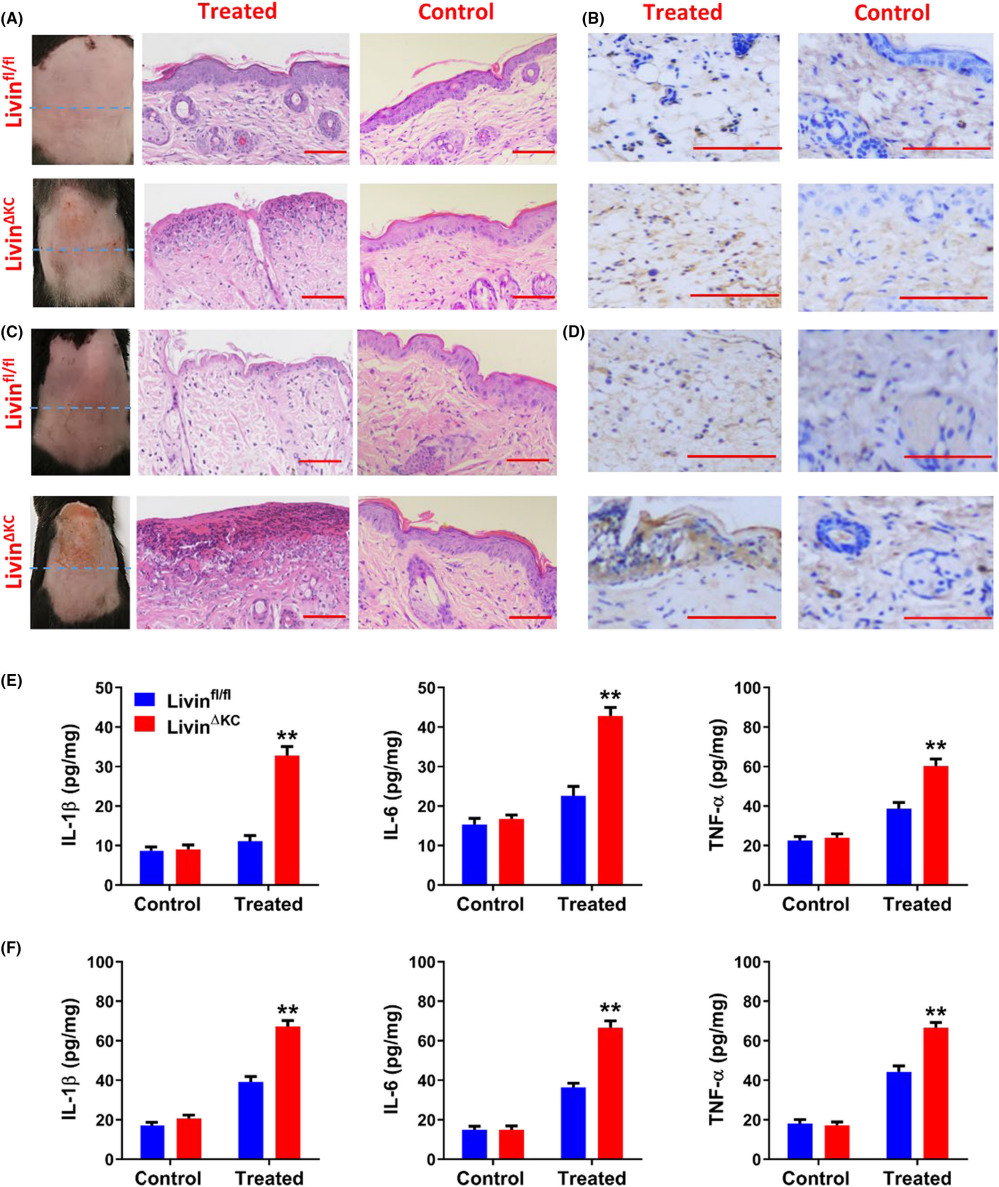
Livin^ΔKC^ mice exhibited more pronounced pathological changes and inflammatory reactions during UVB‐induced skin photodamage. (A, B) Following exposure to 250 mJ/cm^2^ UVB, Livin^fl/fl^ mice displayed no significant pathological changes. In contrast, Livin^ΔKC^ mice showed evident phenotypic alterations, pathological changes, and neutrophil infiltration. (C, D) Upon exposure to 500 mJ/cm^2^ UVB, Livin^fl/fl^ mice experienced notable pathological changes and neutrophil infiltration. However, the impact was much more severe in Livin^ΔKC^ mice, as observed through haematoxylin and eosin staining and immunohistochemical staining at 200× (Panel B) and 400× (Panel D) magnification. (E) The exposure to 250 mJ/cm^2^ of UVB did not induce significant releases of IL‐1β, IL‐6, and TNF‐α in Livin^fl/fl^ mice. In contrast, there were significant elevation in the release of all these cytokines in Livin^ΔKC^ mice. (F) Exposure to 500 mJ/cm^2^ UVB resulted in the release of IL‐1β, IL‐6, and TNF‐α in Livin^fl/fl^ mice. However, Livin^ΔKC^ mice exhibited significantly elevated levels of these cytokines compared to Livin^fl/fl^ mice, as assessed through ELISA analysis. Data are presented as mean ± SEM (*n* = 8/group) and were assessed using two‐way ANOVA. Statistical significance is denoted as ** for *p* < 0.01.

To further analyse the function of Livin in skin photodamage, Livin^ΔKC^ and Livin^fl/fl^ mice were used to induce photodamage by exposure to UVB radiation. The results showed that Livin^ΔKC^ mice exhibited more severe pathological changes and inflammatory reactions during UVB‐induced skin photodamage. After treatment with 250 mJ/cm^2^ UVB, Livin^fl/fl^ mice did not exhibit significant pathological changes whereas Livin^ΔKC^ mice exhibited obvious phenotypic and pathological changes, and neutrophil infiltration (Figure 2A,B). Moreover, 500 mJ/cm^2^ UVB treatment induced significant pathological changes and neutrophil infiltration in Livin^fl/fl^ mice, while Livin^ΔKC^ mice were significantly more severely affected (Figure 2C,D). These results indicate that Livin expression protected the skin against the pathological changes caused by UVB radiation.

UVB‐induced skin photodamage also results in the elevation of IL‐1β, IL‐6 and TNF‐α expression levels. This study examined the levels of the aforementioned cytokines in Livin^ΔKC^ and Livin^fl/fl^ mice. UVB exposure did not lead to significant releases of IL‐1β, IL‐6 and TNF‐α in Livin^fl/fl^ mice, whereas there were substantial releases in Livin^ΔKC^ mice (Figure 2E). Furthermore, following 500 mJ/cm^2^ UVB exposure, low‐level releases of IL‐1β, IL‐6 and TNF‐α were detected in Livin^fl/fl^ mice in comparison with Livin^ΔKC^ mice, which exhibited significantly higher levels than Livin^fl/fl^ mice (Figure 2F).

### 3.3 Analysis of Livin in a mouse model of UVB‐induced skin photodamage using KEGG pathway enrichment

Our previous research has shown the significant involvement of Livin in the development of psoriasis. However, the precise contribution of Livin to KCs in the context of skin photodamage pathogenesis remains unclear. Hence, we employed RNA‐Seq techniques to elucidate the function of Livin in skin photodamage. We examined gene expression differences in UVB‐induced skin photodamage between Livin^ΔKC^ (*n* = 5) and Livin^fl/fl^ mice (*n* = 5). Livin^ΔKC^ mice exhibited 1170 upregulated and 509 downregulated genes in comparison with Livin^fl/fl^ mice (Figure 3A). KEGG pathway enrichment analysis revealed significant associations between Livin and epidermal barrier functions, encompassing *Staphylococcus aureus* infection, cytokine‐cytokine receptor interaction and cell adhesion proteins (Figure 3B). Moreover, GO enrichment analysis indicated that Livin influenced the functions of the epidermis and KCs (Figure 3C).

**Effects of monomethoxypolyethylene glycol‐chitosan nanoparticle‐mediated dual silencing of livin and survivin genes in prostate cancer PC‐3M cellsFIGURE 3:**
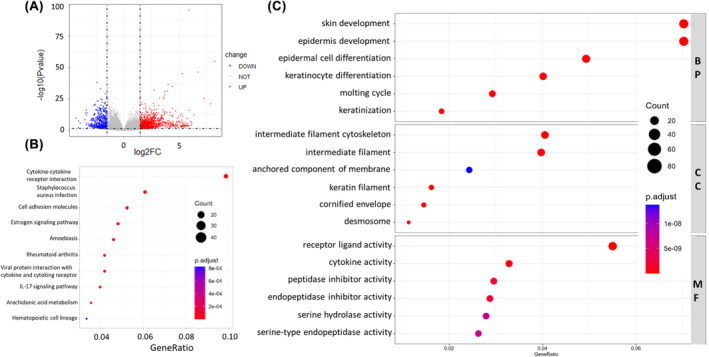
Effects of monomethoxypolyethylene glycol‐chitosan nanoparticle‐mediated dual silencing of livin and survivin genes in prostate cancer PC‐3M cells GO and KEGG pathway enrichment analyses of Livin. (A) When compared with UVB‐treated Livin^fl/fl^ mice, Livin^ΔKC^ mice exhibited 1170 genes with increased expression and 509 genes with decreased expression. (B) KEGG pathway enrichment analysis was conducted on Livin^ΔKC^ mice. (C) GO enrichment analysis was performed on Livin^ΔKC^ mice.

**FIGURE 4:**
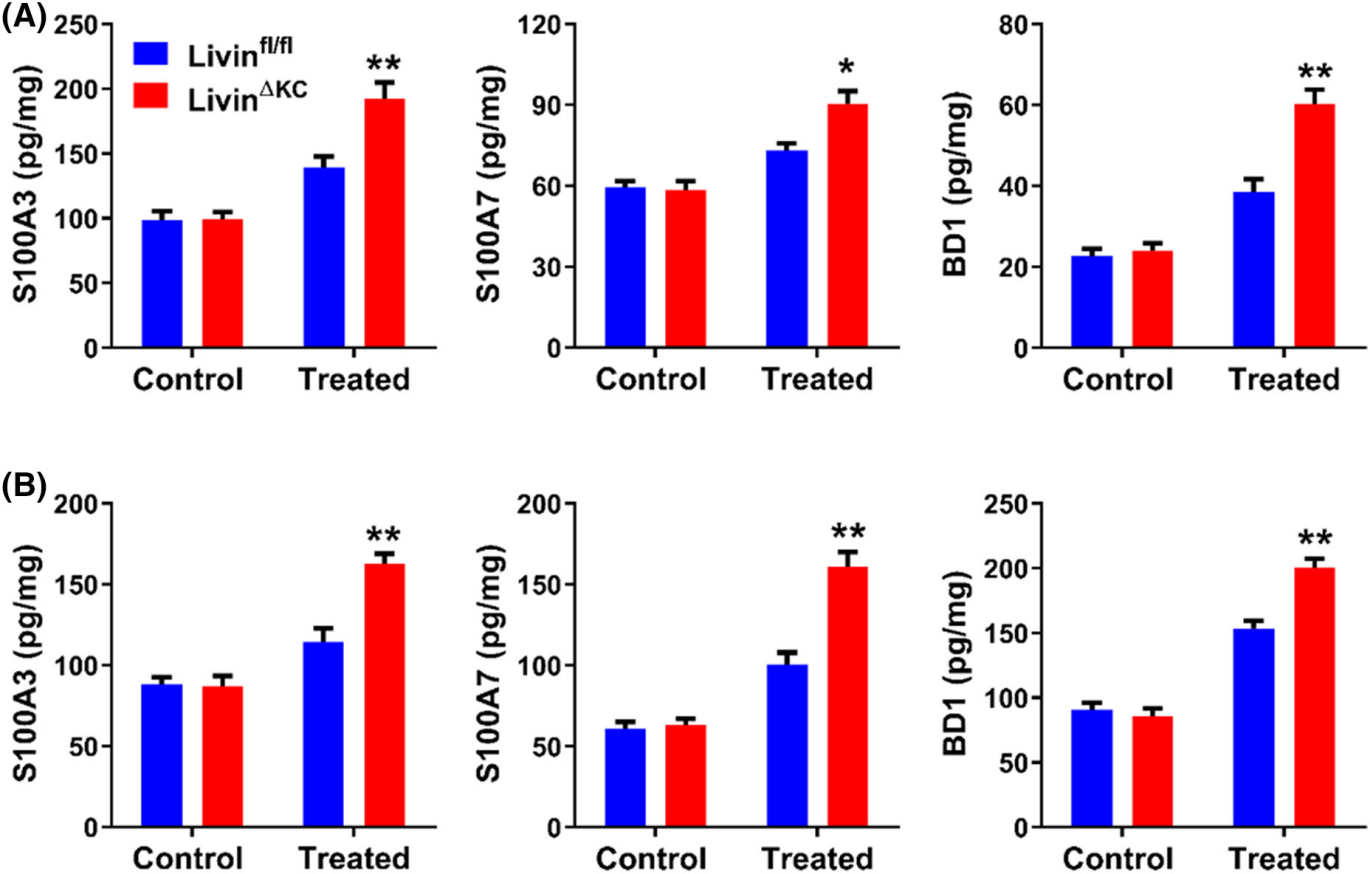
UVB‐treated Livin^ΔKC^ mice released greater quantities of antimicrobial peptides. (A) A lower dose of UVB radiation (250 mJ/cm^2^) did not induce significant releases of S100A3, S100A7 and BD1 in Livin^fl/fl^ mice, whereas there were significant increases in the release of S100A3, S100A7 and BD1 in Livin^ΔKC^ mice. (B) A higher dose of UVB (500 mJ/cm^2^) led to the release of S100A3, S100A7 and BD1 in Livin^fl/fl^ mice, while the releases from Livin^ΔKC^ mice were notably higher than in Livin^fl/fl^ mice, as assessed through ELISA analysis. Data are presented as the mean ± SEM (*n* = 8/group) and were assessed using two‐way ANOVA. Statistical significance is denoted as * for *p* < 0.05 and ** for *p* < 0.01.

Current research suggests that IL‐1β, IL‐6 and TNF‐α play important roles in UVB‐induced damage. However, the study of the inflammatory response in UVB‐induced damage is not complete. After analysing the RNA‐seq data, we discovered that Livin expression influenced the release of antimicrobial peptides such as S100A3, S100A7, and BD1 from KCs, which have been demonstrated to play important roles in many inflammatory skin diseases and could be increased following UVB exposure [16, 17]. UVB exposure at 250 mJ/cm^2^ did not induce significant releases of S100A3, S100A7 or BD1 in Livin^fl/fl^ mice. Conversely, the releases of these peptides were significantly elevated in Livin^ΔKC^ mice (Figure 4A). Upon increasing the UVB dose to 500 mJ/cm^2^, S100A3, S100A7 and BD1 releases were observed in Livin^fl/fl^ mice. However, Livin^ΔKC^ mice exhibited notably higher levels of these peptides than Livin^fl/fl^ mice, as determined by ELISA analysis (Figure 4B).

**FIGURE 5:**
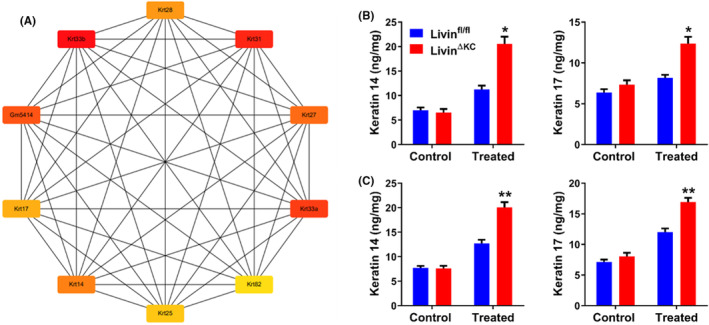
Keratins were significantly upregulated in UVB‐treated Livin^ΔKC^ mice. (A) Protein interaction analysis of UVB‐treated Livin^fl/fl^ and Livin^ΔKC^ mice. (B) A lower dose of UVB radiation (250 mJ/cm^2^) did not induce increases in keratin 14 and keratin 17 levels in Livin^fl/fl^ mice, while there were significant increases in keratin 14 and keratin 17 levels in Livin^ΔKC^ mice. (C) A higher dose of UVB (500 mJ/cm^2^) induced increases in keratin 17 and keratin 14 levels in Livin^fl/fl^ mice, while Livin^ΔKC^ mice exhibited significantly higher levels than Livin^fl/fl^ mice, as assessed through ELISA analysis. Data are presented as the mean ± SEM (*n* = 8/group) and were assessed using two‐way ANOVA. Statistical significance is denoted as * for *p* < 0.05 and ** for *p* < 0.01.

Furthermore, the analysis of protein interactions indicated that Livin expression influenced the expression of keratins (Figure 5A). Keratin 14 and 17 are known to be elevated in UVB‐induced skin photodamage, impacting epidermal barrier function.^16^, ^17^ In this study, UVB exposure at 250 mJ/cm^2^ did not result in elevations in keratin 17 and keratin 14 levels in Livin^fl/fl^ mice. In contrast, these levels were significantly elevated in Livin^ΔKC^ mice (Figure 5B). With an increased UVB dose of 500 mJ/cm^2^, keratin 14 and keratin 17 levels rose in Livin^fl/fl^ mice, whereas Livin^ΔKC^ mice displayed substantially higher levels than Livin^fl/fl^ mice (Figure 5C).

### 3.4 Livin mediated the release of cytokines and the expression of keratin in HaCaT cells damaged by UVB exposure

**FIGURE 6:**
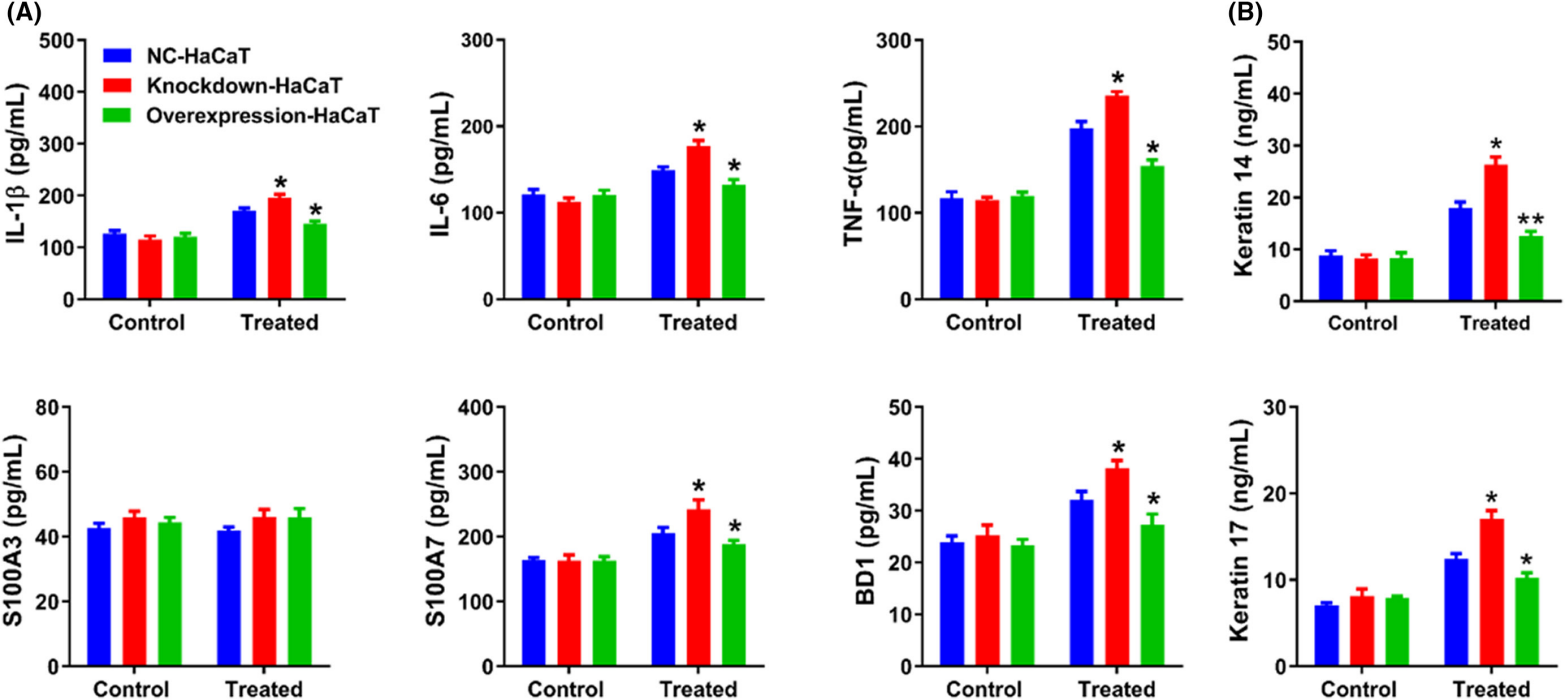
Livin affected cytokine release and keratin expression in HaCaT cells. (A, B) After treatment with UVB radiation (100 mJ/cm^2^), the levels of IL‐1β, IL‐6, TNF‐α, S100A7 and BD1 release, as well as keratin 14 and keratin 17 expression, except S100A3, were increased in Livin knockdown HaCaT cells compared with NC HaCaT cells. Conversely, these indicators were mitigated in Livin overexpressing HaCaT cells compared with NC HaCaT cells, except S100A3. Data are presented as the mean ± SEM and were assessed using one‐ or two‐way ANOVA. Statistical significance is denoted as * for *p* < 0.05, **p < 0.01.

We used NC HaCaT cells, knockdown HaCaT cells and overexpression HaCaT cells to elucidate the role of Livin in HaCaT cells treated with UVB radiation. We examined distinctions among the three groups under standard culture conditions. When not exposed to UVB irradiation, the three groups of cells exhibited minimal differences in the release of IL‐1β, IL‐6, TNF‐α, S100A3, S100A7 and BD1, as well as in the expression of keratin 14 and keratin 17 (Figure S3). However, following exposure to 100 mJ/cm^2^ UVB, IL‐1β, IL‐6, TNF‐α, S100A7 and BD1 releases and keratin 14 and keratin 17 expression levels increased in NC HaCaT cells, with the exception of S100A3. Significantly, the levels in knockdown HaCaT cells showed marked increases, whereas those in overexpressing HaCaT cells exhibited decreases, except S100A3 (Figure 6A,B).

### 3.5 UVB exposure‐induced damage in HaCaT cells and Livin^fl/fl^ mice involves the mediation of NF‐κB activity by Livin

**FIGURE 7:**
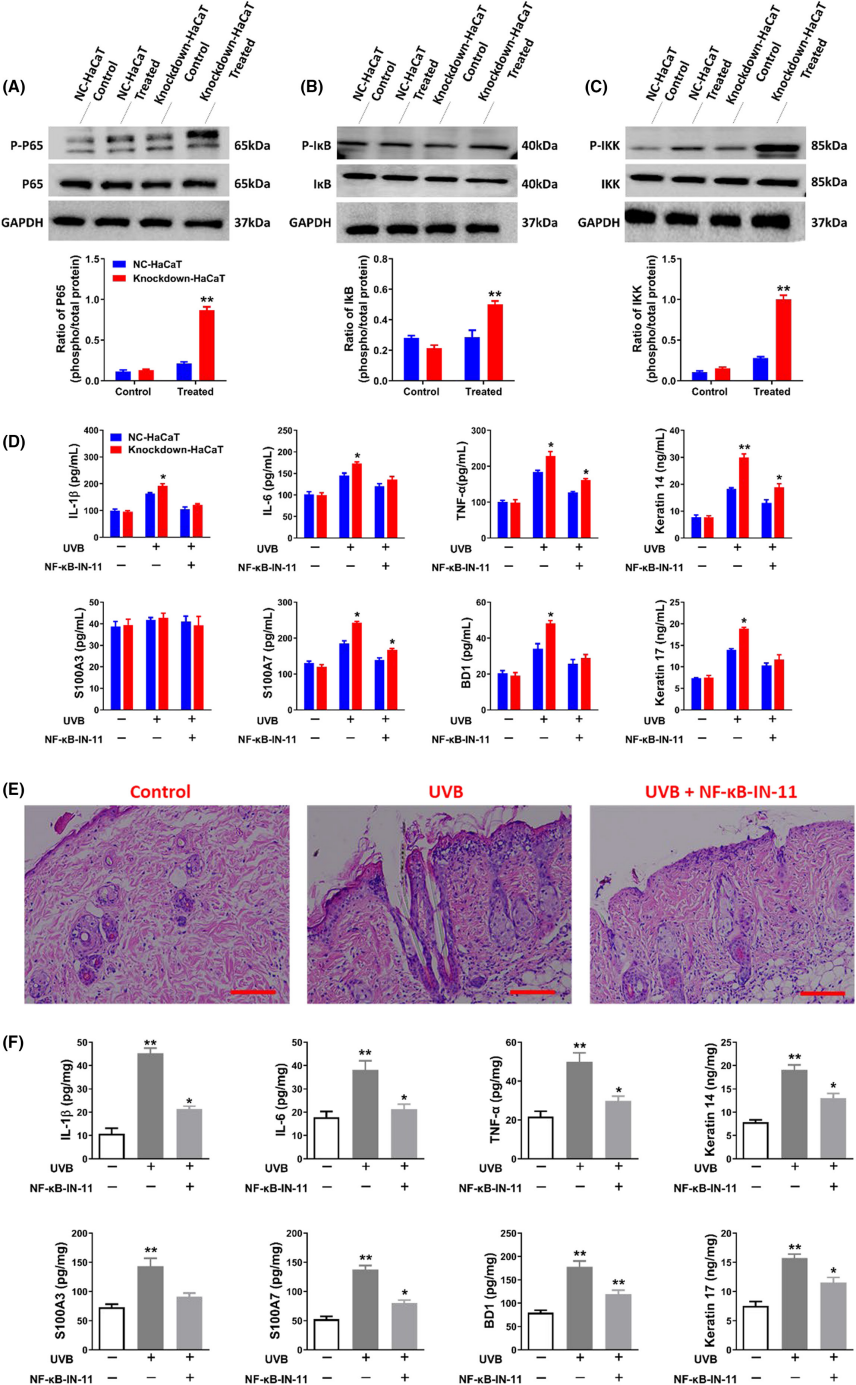
Livin has the potential to trigger NF‐κB activity. **(**A–C) The phosphorylation levels of p65, IκB, and IKK were significantly higher in Livin knockdown HaCaT cells than in negative control HaCaT cells. (D–F) After treatment with NF‐κB‐IN‐11, the levels of IL‐1β, IL‐6, TNF‐α, S100A3, S100A7, and BD1 release and the expression of keratin 14 and keratin 17 were significantly decreased compared with those in the control group both in vivo and in vitro. The results are displayed as the mean ± SEM and were assessed through one‐ or two‐way ANOVA. Significance levels are marked as * for *p* < 0.05 and ** for *p* < 0.01.

In knockdown HaCaT cells, UVB exposure triggered the activation of NF‐κB, a process examined through western blot analysis. UVB treatment activated the NF‐κB cascade, leading to an increase in the phosphorylation level of p65, as well as enhanced phosphorylation of IκB and IκB kinase (IKK). However, after Livin knockdown, in HaCaT cells, the phosphorylation levels of p65, IκB and IKK were notably increased compared to those in NC HaCaT cells (Figure 7A–C). The knockdown of Livin heightened NF‐κB activation. However, an NF‐κB inhibitor (NF‐κB‐IN‐11) was used to inhibit NF‐κB activation in vivo and in vitro. The data showed that after treatment with NF‐κB‐IN‐11, the levels of IL‐1β, IL‐6, TNF‐α, S100A3, S100A7, and BD1 release and the expression of keratin 14 and keratin 17 were significantly decreased compared with those in the control group both in vivo and in vitro (Figure 7D–F).

## 4 DISCUSSION

In our research, we observed a downregulation of the Livin protein in the lesions of patients with skin photodamage. To investigate the function of Livin in the context of skin photodamage, we conducted experiments using Livin^ΔKC^ mice, which have been found to exhibit heightened sensitivity to UVB radiation. UVB exposure amplified the release of inflammatory factors in the mice. Comparatively, Livin^ΔKC^ mice showed more severe skin damage and a greater release of inflammatory factors when exposed to the same UVB dose than Livin^fl/fl^ mice. The reduction in Livin contributed to an increase in keratin expression in Livin^ΔKC^ mice. Livin knockdown induced the stimulation of NF‐κB, subsequently promoting the liberation of inflammatory agents and modulating keratin levels in HaCaT cells. These outcomes suggest that Livin might be involved in the pathogenesis of UVB‐induced photodamage by modulating the NF‐κB signalling cascade in keratinocytes.

Epidermal KCs, the primary target cells of UVB radiation, have a pivotal function in the development of skin photodamage. The exact mechanisms behind skin photodamage induced by UVB radiation are not yet clear and are not completely understood. Past research on these mechanisms has primarily focussed on subjects such as cell apoptosis, DNA damage and repair, and oxidative stress.^18^, ^19^ Nevertheless, while the inflammatory response governed by KCs plays a role in various skin conditions, its contribution to skin photodamage remains uncertain. Furthermore, in our findings, Livin deletion influenced the initiation of NF‐κB. Prior investigations have identified the involvement of the IAP family in both the innate immune response and the stimulation of NF‐κB.^20^ As a recent addition to the IAP family, Livin has been recognized as a participant in regulating inflammation through the NF‐κB cascade in KCs. It is widely acknowledged that NF‐κB has a crucial function in regulating inflammatory responses, and inflammation plays a role in skin photodamage. Our study has substantiated that UVB radiation can induce inflammation in KCs, consequently intensifying the release of cytokines, such as IL‐1β, IL‐6 and TNF‐α. Among these cytokines, TNF‐α, once released by KCs, can potentially trigger neighbouring KCs to enhance their responses. Upon binding with their respective receptors, these cytokines can activate NF‐κB, thereby regulating downstream gene expression.^21^ At the same time, these cytokines can heighten the reaction of skin cells to UV radiation. Inhibiting NF‐κB can diminish the release of cytokines, which serves as a protective measure against UV radiation‐induced skin damage.^22^ Inhibiting the NF‐κB pathway could enhance skin health. Nevertheless, Livin knockdown led to an intensified stimulation of NF‐κB, which could mediate the increased sensitivity of Livin‐deficient mice to UVB‐induced skin photodamage. Likewise, our cellular experiments confirmed the role of Livin in regulating inflammatory responses.

The conclusions drawn from this current study suggest that Livin demonstrates a certain level of safeguarding impact against UVB radiation‐induced skin photodamage. Livin deficiency heightened the inflammatory response in KCs following exposure to UVB radiation in mouse skin photodamage models. Based on the current study, Livin could serve as a target for the development of novel drug therapies for managing skin photodamage. In future, experiments aimed at upregulating Livin expression in animals could offer insights into the translational significance of Livin in the context of skin photodamage.

## AUTHOR CONTRIBUTIONS

**Kaijie Wang:** Formal analysis (equal); investigation (equal); methodology (equal); writing – original draft (equal). **Xiaolan You:** Software (equal). **Zhenri Qu:** Resources (equal). **Delu Che:** Formal analysis (equal); methodology (equal); project administration (equal); writing – review and editing (equal). **Xianwei Cao:** Conceptualization (equal); writing – review and editing (equal).

## CONFLICT OF INTEREST STATEMENT

The authors have no conflicts of interest to disclose.

## INFORMED CONSENT

The patients had signed informed consents for the use of their skin tissue specimens.

## Supporting information


Figures S1–S3.

## Data Availability

Original RNA‐seq data have been submitted to BioProject ID PRJNA993569. All the necessary data to validate the conclusions presented in the paper are available either within the paper itself or in the Supplementary Materials. The original data are available from the corresponding author upon reasonable request.

## References

[1] Heng M . Wound healing in adult skin: aiming for perfect regeneration. Int J Dermatol. 2011;50(9):1058‐1066. 22126865 10.1111/j.1365-4632.2011.04940.x

[2] Wang S , Yang M , Yin S , et al. A new peptide originated from amphibian skin alleviates the ultraviolet B‐induced skin photodamage. Biomed Pharmacother. 2022;150:112987. 35462334 10.1016/j.biopha.2022.112987

[3] Biswas S , Mukherjee PK , Kar A , et al. Enhanced permeability and photoprotective potential of optimized p‐coumaric acid‐phospholipid complex loaded gel against UVA mediated oxidative stress. J Photochem Photobiol B. 2021;21:112246. 10.1016/j.jphotobiol.2021.112246 34243023

[4] Panich U , Sittithumcharee G , Rathviboon N , Jirawatnotai S . Ultraviolet radiation‐induced skin aging: the role of DNA damage and oxidative stress in epidermal stem cell damage mediated skin aging. Stem Cells Int. 2016;2016:7370642. 27148370 10.1155/2016/7370642 PMC4842382

[5] Park YK , Jang BC . UVB‐induced anti‐survival and pro‐apoptotic effects on HaCaT human keratinocytes via caspase‐ and PKC‐dependent downregulation of PKB, HIAP‐1, Mcl‐1, XIAP and ER Stress. Int J Mol Med. 2014;33(3):695‐702. 24356997 10.3892/ijmm.2013.1595

[6] Gasparrini M , Forbes‐Hernandez TY , Afrin S , et al. Strawberry‐based cosmetic formulations protect human dermal fibroblasts against UVA‐induced damage. Nutrients. 2017;9(6):605. 28613256 10.3390/nu9060605 PMC5490584

[7] Syed DN , Khan MI , Shabbir M , Mukhtar H . MicroRNAs in skin response to UV radiation. Curr Drug Targets. 2013;14(10):1128‐1134. 23834148 10.2174/13894501113149990184 PMC3985496

[8] Wang ML , Zhong QY , Lin BQ , Liu YH , Huang YF . Andrographolide sodium bisulfate attenuates UV‐induced photo‐damage by activating the keap1/Nrf2 pathway and downregulating the NF‐κB pathway in HaCaT keratinocytes. Int J Mol Med. 2020;45(2):343‐352. 31789424 10.3892/ijmm.2019.4415 PMC6984792

[9] Yang AQ , Wang PJ , Huang T , Zhou WL , Landman J . Effects of monomethoxypolyethylene glycol‐chitosan nanoparticle‐mediated dual silencing of livin and survivin genes in prostate cancer PC‐3M cells. Genet Mol Res. 2016;15(2):7430. 10.4238/gmr.15027430 27173182

[10] Cho SB , Lee WS , Park YL , et al. Livin is associated with the invasive and oncogenic phenotypes of human hepatocellular carcinoma cells. Hepatol Res. 2015;45(4):448‐457. 24934632 10.1111/hepr.12374

[11] Che D , Hang B , Li Y , Li K , Wang K , Wang H . Livin upregulation in keratinocytes of psoriasis patients to promote adhesion molecule expression. Int J Dermatol. 2023;62(7):900‐909. 36916467 10.1111/ijd.16621

[12] Sklar LR , Almutawa F , Lim HW , Hamzavi I . Effects of ultraviolet radiation, visible light, and infrared radiation on erythema and pigmentation: a review. Photochem Photobiol Sci. 2013;12(1):54‐64. 23111621 10.1039/c2pp25152c

[13] Rybchyn MS , De Silva WGM , Sequeira VB , et al. Enhanced repair of UV‐induced DNA damage by 1,25‐Dihydroxyvitamin D3 in skin is linked to pathways that control cellular energy. J Invest Dermatol. 2018;138(5):1146‐1156. 29258892 10.1016/j.jid.2017.11.037

[14] Averilla JN , Oh J , Kim JS . Carbon monoxide partially mediates protective effect of resveratrol against UVB‐induced oxidative stress in human keratinocytes. Antioxidants (Basel). 2019;8(10):432. 31581413 10.3390/antiox8100432 PMC6827139

[15] Gendrisch F , Esser PR , Schempp CM , Wölfle U . Luteolin as a modulator of skin aging and inflammation. Biofactors. 2021;47(2):170‐180. 33368702 10.1002/biof.1699

[16] Marcinkiewicz M , Majewski S . The role of antimicrobial peptides in chronic inflammatory skin diseases. Postepy Dermatol Alergol. 2016;33(1):6‐12. 26985172 10.5114/pdia.2015.48066 PMC4793058

[17] Morio KA , Sternowski RH , Zeng E , Brogden KA . Antimicrobial peptides and biomarkers induced by ultraviolet irradiation have the potential to reduce endodontic inflammation and facilitate tissue healing. Pharmaceutics. 2022;14(9):1979. 36145725 10.3390/pharmaceutics14091979 PMC9503046

[18] Farooqi AA , Li RN , Huang HW , et al. Natural products mediated regulation of oxidative stress and DNA damage in ultraviolet exposed skin cells. Curr Pharm Biotechnol. 2015;16(12):1078‐1084. 26238680 10.2174/1389201016666150731111308

[19] Matsumura Y , Ananthaswamy HN . Toxic effects of ultraviolet radiation on the skin. Toxicol Appl Pharmacol. 2004;195(3):298‐308. 15020192 10.1016/j.taap.2003.08.019

[20] Cartier J , Marivin A , Berthelet J , Dubrez L . Les IAP au cœur de la signalisation NF‐κB [IAPs: a central element in the NF‐κB activating signaling pathway]. Med Sci (Paris). 2012;28(1):69‐75. 22289833 10.1051/medsci/2012281019

[21] Farag AGA , Hammam MA , Al‐Sharaky DR , El‐Boghdady GM . Leucine‐rich glioma inactivated 3: a novel keratinocyte‐derived melanogenic cytokine in vitiligo patients. An Bras Dermatol. 2019;94(4):434‐441. 31644616 10.1590/abd1806-4841.20198250 PMC7007044

[22] Tanaka K , Asamitsu K , Uranishi H , et al. Protecting skin photoaging by NF‐kappaB inhibitor. Curr Drug Metab. 2010;11(5):431‐435. 20540695 10.2174/138920010791526051

