# Supplementary material for: Livin is protective in UVB‐induced skin photodamage by regulating keratinocyte activation and inflammatory responses
Source: J Cell Mol Med. 2024 Feb 8;28(4):e18124. doi: 10.1111/jcmm.18124 (PMC10853578; doi:10.1111/jcmm.18124)
Supplement: Supplementary file 1 — Figures S1–S3. [file JCMM-28-e18124-s001.docx]

**
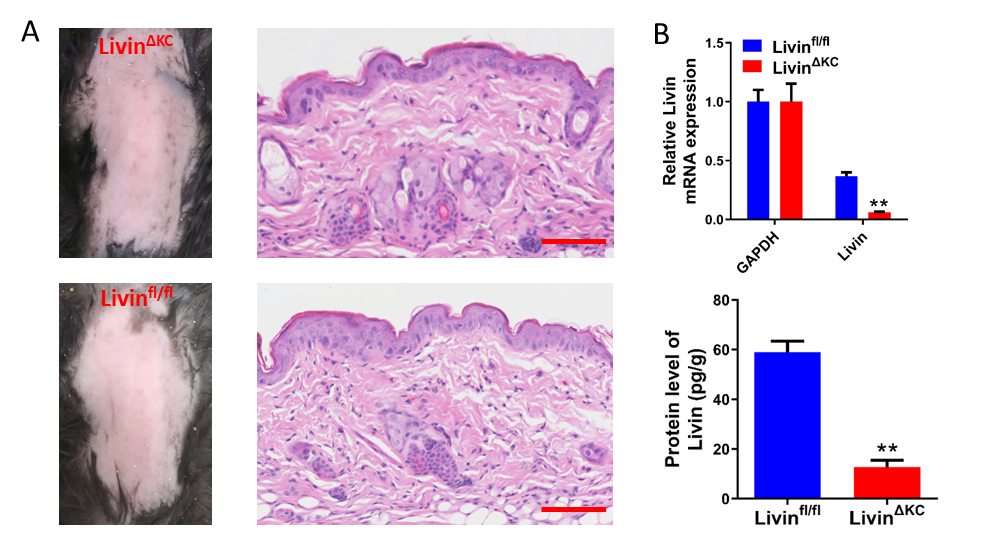
**

**Supplementary Figure. 1.** Livin expression analysis in Livin^ΔKC^ mice. A: Livin^ΔKC^ mice showed little difference than Livin^fl/fl^ mice in phenotype and showed no significant pathological changes analyzed by H&E staining. B: Livin expression was significant decrease in Livin^ΔKC^ mice analyzed by RT-qPCR and ELISA. Data were presented as mean ± SEM (n=8/group) and were assessed using Student’s t test. Statistical significance is denoted as ** for p＜0.01.


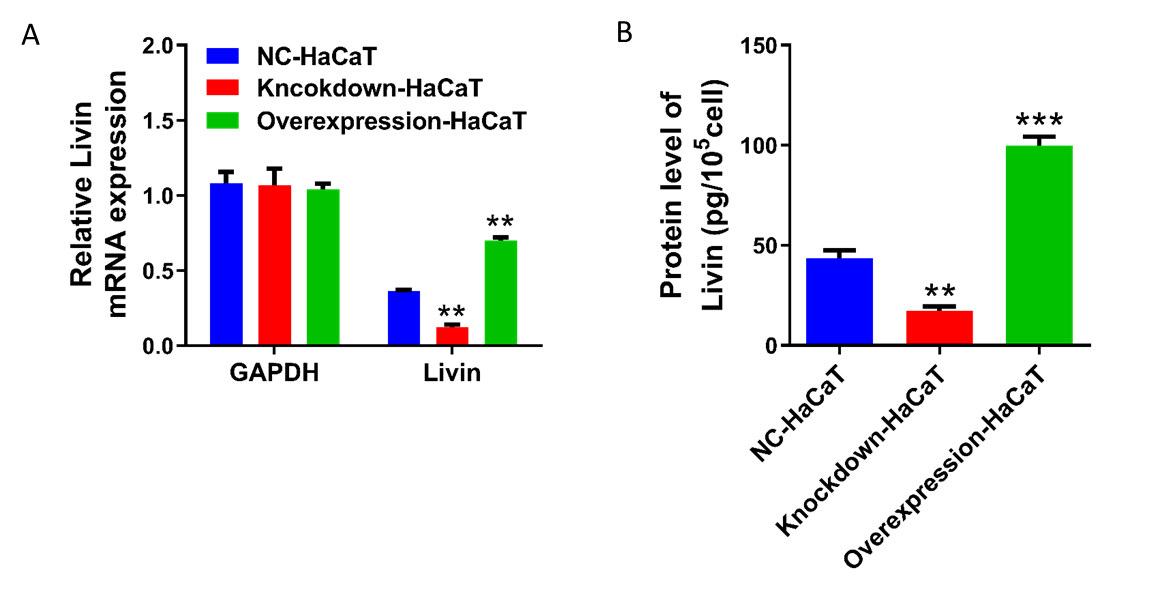


**Supplementary Figure. 2.** Livin expression in NC HaCaT cells, knockdown HaCaT cells and overexpression HaCaT cells. A–B: Livin expression analyzed by RT-qPCR and ELISA. Data were presented as mean ± SEM and were assessed using one-way ANOVA. Statistical significance is denoted as ** for p＜0.01 and *** for p＜0.001.


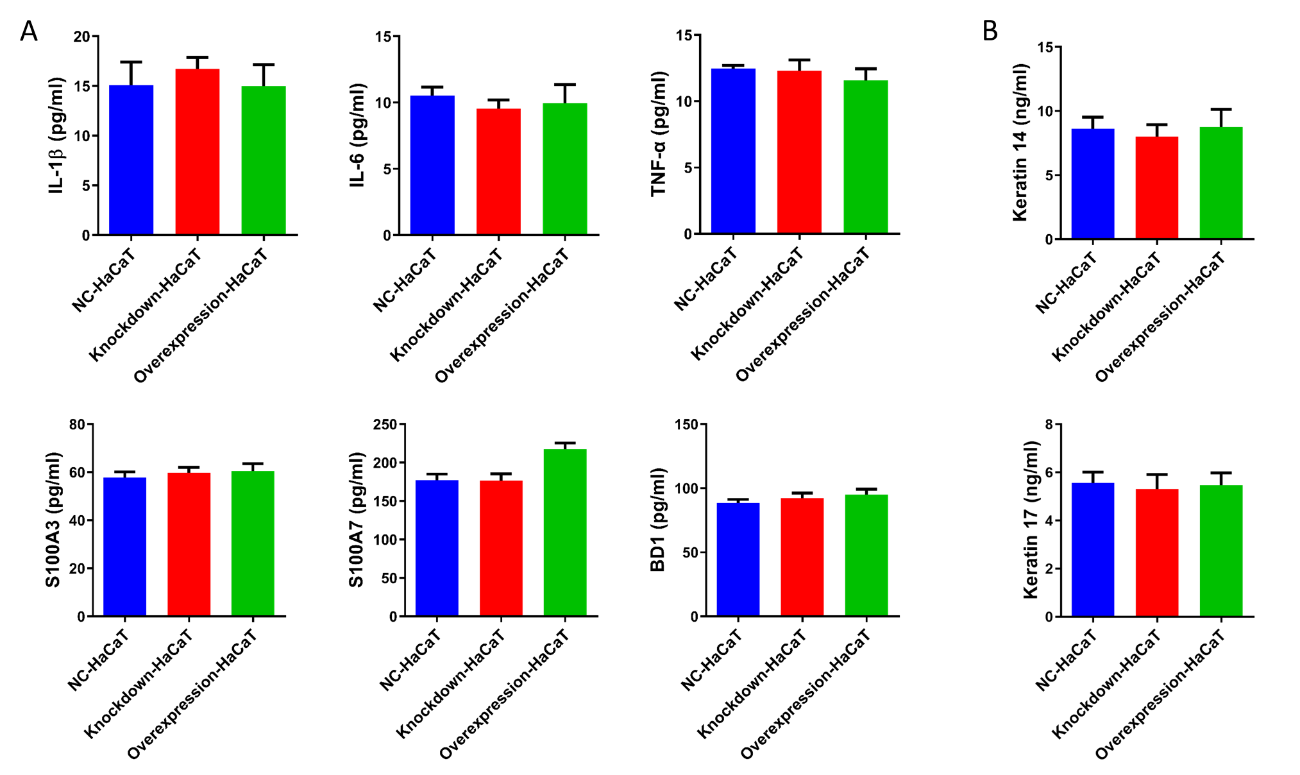


**Supplementary Figure. 3.** Livin affected cytokine release and keratin expression in HaCaT cells. A–B: In the absence of UVB irradiation, no notable distinction was observed among NC HaCaT cells, Livin knockdown HaCaT cells, and Livin overexpressing HaCaT cells regarding the release of IL-1β, IL-6, TNF-α, S100A3, S100A7, and BD1 and the expression of keratin 14 and keratin 17 under normal culture conditions. Data are presented as the mean ± SEM and were assessed using one- or two-way ANOVA.
